# Parental Attachment and Problematic Internet Use among Chinese Adolescents: The Moderating Role of Gender and Grit

**DOI:** 10.3390/ijerph17238933

**Published:** 2020-12-01

**Authors:** Xiaoyu Lan, Wenchao Wang

**Affiliations:** Beijing Key Laboratory of Applied Experimental Psychology, Faculty of Psychology, Beijing Normal University, Beijing 100875, China; xiaoyu.lan@phd.unipd.it

**Keywords:** problematic Internet use, parental attachment, gender, grit, Chinese adolescents

## Abstract

Problematic Internet use (PIU) is currently becoming a more serious public health concern, representing a deleterious effect on adolescent adaptive emotional and behavioral patterns. Given the prevalence of PIU and its deleterious impact on adolescents’ optimal functioning, it is valuable to investigate the risk and protective factors of PIU. Guided by a socio-ecological framework, the current study examines the associations of paternal attachment and maternal attachment with PIU among Chinese adolescents. Furthermore, this study investigates whether adolescents’ gender and grit moderate this association. A total of 2677 Chinese adolescents (56.5% girls; *M*_age_ = 15.56; *SD* = 1.57) was involved in this study. Adolescents were uniformly instructed to complete a battery of self-reported questionnaires. The results of linear regression analyses showed that paternal attachment and maternal attachment security were negatively related to PIU. Moreover, moderation analyses revealed that higher levels of grit buffered against boys’ PIU in the context of paternal attachment security and girls’ PIU in the context of paternal attachment insecurity. The current study suggests that parental attachment security plays an important role in mitigating the likelihood of Chinese adolescents’ PIU. Moreover, the buffering role of grit in PIU varies by the levels of paternal attachment security, depending on the adolescents’ gender.

## 1. Introduction

With the rapid economic growth in mainland China, Internet use has dramatically increased in the last few decades. Based on a recent report from the China Internet Network Information Center (2019), the Internet has penetrated 57.6% of China’s total population, and there has been an increase of 56 million Internet users over the last year. The growth in Internet use has been paralleled by emerging concerns about problematic Internet use (PIU). As a general behavioral addiction, PIU refers to a maladaptive preoccupation with using the Internet, which leads to significant distress or impairment resulting from the behavior [1,2,3]. The likelihood of developing PIU is more eminent during adolescence, as youths rapidly adopt new technologies, but their cognitive control abilities are relatively immature [4,5,6]. As noted by previous studies in Chinese adolescents [4,7], PIU is currently becoming a more serious public health concern. In this context, the onset of PIU could interfere with adolescents’ daily life, leading to a plethora of psychosocial adversities and underachievement in school [8,9]. Given the prevalence of PIU and its deleterious impact on adolescent adaptive emotional and behavioral patterns, it is necessary to investigate the risk and protective factors of PIU among them.

To investigate the risk and protective factors of PIU, we refer to the socio-ecological theory as an overarching theoretical framework [10]. According to this theory, behaviors evolve on a continuum due to the interaction of contextual and personal factors [11]. Considering PIU in particular, addictions are the result of socio-ecological processes that involve the effects of contextual and personal characteristics and their dynamic interactions [12]. The socio-ecological framework has been successfully applied to investigate the risk and protective factors in adolescents’ PIU [13]. In the current study, we focus on parental attachment as a contextual factor and adolescent’s gender and grit as individual characteristics, documenting how these factors directly and interactively influence Chinese adolescents’ PIU. In the following paragraphs, we conduct a literature review concerning each of the study variables, starting from the presentation of parental attachment and gender differences.

### 1.1. Parental Attachment and Gender Differences

Attachment refers to a long-lasting emotional bond established between children and their primary caregivers during early childhood [14]. According to attachment theory [14,15], interactions with attachment figures are stored in their mental representations based on internal working models of self and others. The internal working models that children develop are carried forward into adolescence and form the basis of their social relationships, which, in turn, impacts their well-being [14,15]. For example, adolescents with attachment security have the internal working models of self as worthy of love and support and others as reliable and responsive [15,16]. In this perspective, attachment figures’ sensitive and responsive care could soothe adolescent psychological distress and alleviate the possibility of adolescents’ PIU; on the contrary, attachment figures’ irresponsiveness and unavailability potentially increase the likelihood of adolescents’ PIU.

As documented by a myriad of empirical studies, attachment insecurity is identified as a risk factor for PIU in adolescents [17,18,19], also in Chinese adolescents [20]. This is because adolescents with parental attachment insecurity are likely to overuse the Internet in an attempt to seek comfort and belongingness [19]. Despite these research advances, much of the research highlights the importance of maternal attachment only or employing an overall parental attachment score. However, little is known about the possible differing roles of paternal and maternal attachment in adolescents’ PIU. In response to this knowledge gap, a mounting body of research has exhibited the indispensable role of Chinese fathers in adolescent psychological health and adaptive behavioral patterns [21,22,23]. Given that fathers and mothers may play different roles in adolescent behavioral development, it is essential to simultaneously examine the roles of paternal attachment and maternal attachment in adolescents’ PIU.

Furthermore, little research attention has been devoted to the possibility of a gender difference in the association between parental attachment and adolescents’ PIU; that is, whether paternal attachment and maternal attachment may exert different influences on boys’ and girls’ PIU. Prior research based on a sample of undergraduate students in the U.S. has shown that insecure paternal attachment leads to PIU for girls, whilst insecure maternal attachment contributes to PIU for boys, highlighting the opposite gender patterns in the onset of PIU [24]. Despite this initial evidence, it is still unclear how these association patterns are exhibited in Chinese adolescents. Attempting to address this unfilled gap, it is meaningful to explore whether an opposite/same gender pattern between paternal/maternal attachment and adolescents’ gender is exhibited in Chinese adolescents’ PIU. Apart from the gender difference, the role of individual characteristics, such as grit, in the link between parental attachment and PIU is also considered.

### 1.2. Grit

Grit refers to continuous perseverance and passion for long-term goals despite failures and obstacles [25]. We focus on the role of grit in the current study based on the following considerations. Theoretically, Bronfenbrenner’s socio-ecological theory highlights that adolescents’ development occurs in the context of multiple interacting environmental systems [10]. Research has increasingly recognized grit as one of the important individual characteristics in facilitating adolescent adaptive behavioral patterns [26,27,28,29]. Culturally, as delineated by a Chinese proverb, “it takes more than one cold day for a river to freeze a meter deep”, diligence, perseverance, and determination are highly emphasized in traditional Chinese culture [26,27]. Given these theoretical and cultural emphases of grit on adaptive behavioral socialization, it is important to address the role of grit in adolescents’ PIU.

Recently, a few empirical studies using a sample of Chinese adolescents have shown that grit is positively associated with adolescent adaptive functions [29,30,31]. In terms of PIU, prior research using a sample of undergraduate students exhibited that grit is negatively associated with PIU [32]. Despite this finding, relatively little is known about how grit is related to PIU in adolescence. Highly relevant to the current study, Li and Zhu (2020) reported that grit moderates the association between peer victimization and problematic Internet game use [31]. More precisely, peer victimization serves as a significant risk factor for problematic Internet game use among adolescents with lower grit levels, but not for adolescents with higher grit. The authors explained that gritty adolescents tend to have high self-efficacy and strong desires to mobilize all available resources to overcome difficulties in front of adversities. Informed by this initial evidence, we propose that grit may also moderate the association between parental attachment and PIU among Chinese adolescents.

### 1.3. Overview and Hypotheses

The present study addresses prior research limitations by investigating the associations of paternal attachment and maternal attachment with PIU and examining whether gender and grit moderate this association among a large-scale sample of Chinese adolescents. Specifically, we examine the following hypotheses:

**Hypothesis 1 (H1).**  *Paternal attachment security and maternal attachment security are negatively associated with PIU.*

**Hypothesis 2 (H2).**  *Paternal attachment security is strongly and negatively related to girls’ PIU, while maternal attachment security is strongly and negatively linked to boys’ PIU (2a); moreover, these associations may be enhanced in higher levels of grit than lower levels of grit (2b).*

It should be noted that although uncovering the moderating role of gender and grit in the link between parental attachment and PIU has been supported by both theoretical and empirical evidence [10,21,33], the second hypothesis is more exploratory in nature (than confirmatory) due to the complexity of three-way interaction terms and the scarcity of prior knowledge on these associations.

Moreover, prior research has provided substantial evidence regarding the links between adolescents’ PIU and sociodemographic factors, such as adolescents’ age and family socioeconomic status (SES) [34,35], although the research findings are somehow contradictory. For instance, some scholars report that family income/parental education is positively linked to adolescents’ PIU, while others show a negative link between them [35]. To increase the robustness of the current findings, we consider adolescents’ age and family SES as covariates that are statistically controlled in the analyses. Nevertheless, given the mixed results in the extant literature, we do not generate specific hypotheses about the associations of age and family SES with PIU.

## 2. Method

### 2.1. Participants and Procedures

Before data collection, this study was ethically approved by the Institutional Review Board of the Beijing Normal University (protocol # 2673). Through personal networks and school collaborations, we sought the school institutions’ help for access adolescents from Grade 7 to Grade 11 in urban settings. After obtaining permission from school principals, we asked head teachers in each classroom to send the informed consent to parents through the Parent WeChat Group. In the meantime, we asked the adolescents whether they were willing to participate in the present research. Both parents and adolescents were assured of the voluntary and confidential nature of this study. After confirming the consent, trained research assistants provided standardized instructions and administered this investigation in the classrooms during a regular class hour (i.e., approximately 45 min). Adolescents were asked to complete a set of self-report questionnaires independently, and these questionnaires were administered in paper-and-pencil format and written in simplified Chinese. Once completed, adolescents were required to return the questionnaires to trained research assistants promptly. At the end of this investigation, adolescents received stationery gifts to thank them for their participation [36]. Finally, a total of 2677 Chinese adolescents (56.5% girls) aged from 13 to 18 years (*M*_age_ = 15.56; *SD* = 1.57) was involved in this study.

### 2.2. Measures

#### 2.2.1. Problematic Internet Use

Problematic Internet use was assessed by the Chinese adaption of the Diagnostic Questionnaire for Internet Addiction [8,37]. This questionnaire was developed based on the Diagnostic and Statistical Manual of Mental Disorders (DSM-IV) criteria. Participants were asked to indicate whether they had experienced each statement or not on a dichotomous scale (0 = no, 1 = yes). This questionnaire consists of 8 items, and one item example is, “Do you feel restless, moody, depressed, or irritable when attempting to cut down or stop Internet use?”. According to prior research [8,37], a sum score of all these items was calculated, with higher scores indicating more severe PIU. Prior research has shown the good internal consistency of this questionnaire in Chinese adolescents [8]. In the current study, Cronbach’s alpha was 0.78.

#### 2.2.2. Parental Attachment

Parental attachment was measured using the short form of the Inventory of Parent Attachment [38]. This inventory has been previously validated in Chinese adolescents by Zhang, Zhang, Zhang, Wang, and Hung (2011), showing good psychometric properties [39]. This inventory contains 30 items (15 items for paternal attachment and 15 items for maternal attachment), capturing three attachment domains (i.e., trust, communication, and alienation). One of the example items is “My father/mother respects my feeling.” Participants were asked to assess the quality of their relationships with fathers/mothers on a 5-point Likert-type ranging from 1 (never true) to 5 (always true). Scores related to each parent were averaged to yield a paternal and a maternal attachment score, with higher scores indicating more secure attachment to fathers/mothers. These scores reflected high trust and communication with fathers/mothers and a low sense of alienation from fathers/mothers [40]. Previous studies have exhibited the good internal consistency of this inventory in Chinese adolescents [23]. In the current study, Cronbach’s alpha was 0.90 for both paternal and maternal attachment.

#### 2.2.3. Grit

Grit was assessed using the Grit Scale [41]. This scale consists of 12 items, and one item example is, “setbacks do not discourage me.” Participants were asked to rate each item from 1 (not like me at all) to 5 (very much like me) on a Likert-type scale. The average score of all items was calculated, with a higher score indicating a higher grit level. This scale has shown good internal consistency based on prior research in Chinese adolescents [27,28]. In this study, Cronbach’s alpha was 0.80.

#### 2.2.4. Sociodemographic Information

Adolescents were asked to indicate their sociodemographic information, including gender, age, parental educational level, parental occupational status, and family monthly income. As suggested by previous studies [42,43], we first standardized the scores of parental educational level, parental occupational status, and family monthly income. Second, these standardized scores were summarized to represent the overall score of family SES.

### 2.3. Data Analyses

We used SPSS 21.0 to perform the data analyses [44]. Before the data analyses, exploratory analyses omitted eighty cases with a higher level of missing data (more than 20%), yielding a final sample of 2677 adolescents. To evaluate the influence of remaining missing data (less than 20%), Little’s Missing Completely at Random (MCAR) test was performed. The findings supported the MCAR assumption, χ^2^(48) = 50.98, *p* = 0.36. Hence, the remaining missing values were imputed according to each participant’s mean value on the corresponding measure [45,46].

Descriptive information was summarized using means and standard deviations for continuous variables, and Pearson’s correlation analyses were used to evaluate associations among study variables. All these analyses were separated by boys and girls. We used a series of independent sample *t*-tests to assess between-group (i.e., adolescents’ gender) differences in study variables.

To test our research hypotheses, we applied multiple linear regression analyses using maximum likelihood estimation. As the preliminary analysis revealed a strong correlation between paternal attachment and maternal attachment (*r* = 0.70, *p* < 0.001; see Table 1), we conducted two linear regression models, separated for each statistical predictor (i.e., paternal attachment and maternal attachment), to avoid multicollinearity issues [47]. In each linear regression model, key study variables (i.e., paternal/maternal attachment, gender, and grit) and interaction terms between these study variables, as well as covariates were entered simultaneously. In this step of the analyses, the categorical variable (i.e., gender) was dummy coded as 1 for boys and 2 for girls, and continuous variables were centered using the grand mean before creating the interaction terms [48]. As suggested by prior research [49], we report the unstandardized beta (*b*), the standard error for the unstandardized beta (*SE b*), the 95% confidence intervals for the unstandardized beta (95 CI for *b*), the *t*-test statistic (*t*), and the probability value (*p*) for each linear regression model. Moreover, R^2^ was used to interpret the percentage of variance explained by all study variables. In addition, to probe the nature of significant interactions, we performed a simple slope analysis [50]. The significant interactions were further interpreted by different sample-estimated values of the moderator (i.e., the moderator’s mean and ±1 standard deviation from the moderator’s mean) and corresponding figures [51]. When describing the interactive figures, −1 standard deviation from the variable’s mean, the variable’s mean, and +1 standard deviation from the variable’s mean were regarded as low, medium, and high levels of the variables. In all these analyses mentioned above, the significance level was interpreted at *p* < 0.05 using two-tailed tests, and the 95% confidence intervals should not contain zeros [52].

## 3. Results

### 3.1. Descriptive Statistics and Correlation Analyses

Descriptive statistics and correlations among study variables are reported in Table 1, separately for boys and girls. As shown in Table 1, for boys, paternal attachment, maternal attachment, and grit were each significantly and negatively associated with PIU, but no significant correlations were found between covariates (i.e., age and family SES) and PIU. By contrast, these correlation patterns mentioned above were also replicated for girls.

Table 1 also presents the results of independent samples of *t*-tests of the study variables between genders. Boys reported higher scores in grit and PIU, but lower scores in maternal attachment than girls.

### 3.2. Associations of Parental Attachment, Adolescents’ Gender, and Grit with PIU

As shown in Table 2, when paternal attachment was regarded as the independent variable, the linear regression model explained 12.2% of the variance in PIU. To be specific, paternal attachment and grit were negatively associated with PIU, but no significant associations of age and family SES with PIU were found. In this step of the analysis, gender was dummy coded, and girls were compared with the reference group (i.e., boys). The negative regression coefficient of the gender indicated that boys reported higher scores of PIU than girls. Moreover, the two-way interaction between adolescents’ gender and grit was positively associated with PIU, but other two-way interactions were not significantly related to PIU. It is noteworthy that in this study, we established lower levels of the interaction terms (i.e., two-way interactions) sequentially to create the three-way interaction term, although the interaction between the two moderators (i.e., gender and grit) was not our research focus [33]. Therefore, in this study, we did not further interpret this significant two-way interaction. In addition, the three-way interaction among paternal attachment, adolescents’ gender, and grit was positively related to PIU.

**Figure 1:**
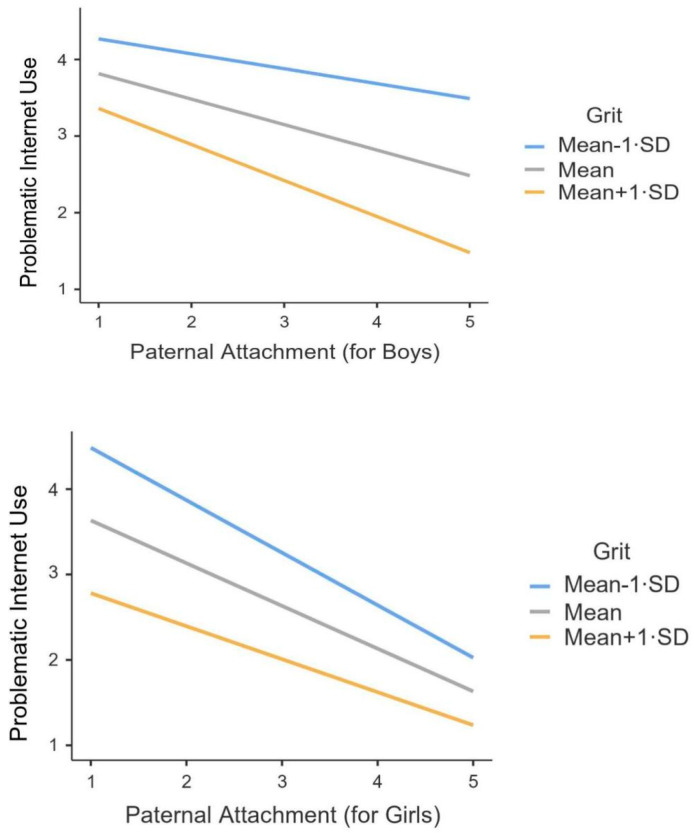
Interaction effect of paternal attachment, adolescents’ gender, and grit on problematic Internet use. Notes: *N* = 2677. Grit was divided into three levels based on the mean: *M* − 1 *SD*, *M*, and *M* + 1 *SD*.

Follow-up simple slope analysis exhibited that for boys, the negative association between paternal attachment and PIU was significant at mean (*b* = −0.33, *SE* = 0.09, *t* = −3.65, *p* < 0.001) and higher levels of grit (*b* = −0.47, *SE* = 0.11, *t* = −4.16, *p* < 0.001), but not at lower levels of grit (*b* = −0.19, *SE* = 0.12, *t* = −1.55, *p* = 0.12). For girls, this negative association between paternal attachment and PIU was significant across lower (*b* = −0.61, *SE* = 0.09, *t* = −6.32, *p* < 0.001), mean (*b* = −0.50, *SE* = 0.07, *t* = −6.62, *p* < 0.001), and higher levels of grit (*b* = −0.38, *SE* = 0.09, *t* = −3.88, *p* < 0.001; see Figure 1). From a descriptive point of view, for boys, in the context of paternal attachment security, higher levels of grit significantly buffered against the levels of PIU; by contrast, in the context of paternal attachment insecurity, the levels of grit seemed independent of the scores for PIU. For girls, in the context of paternal attachment security, the levels of grit seemed independent of the scores for PIU; by contrast, in the context of paternal attachment insecurity, higher levels of grit significantly buffered against the scores for PIU.

Furthermore, as shown in Table 3, when maternal attachment was regarded as the independent variable, the linear regression model explained 12.5% of the variance in PIU. To be specific, maternal attachment and grit were negatively associated with PIU; boys reported higher scores of PIU than girls. However, no significant interactions among study variables were found.

## 4. Discussion

This study aims to simultaneously examine the associations of paternal attachment and maternal attachment with PIU among a large-scale sample of Chinese adolescents. Moreover, this study investigates whether adolescents’ gender and grit moderate this association. The current findings exhibit that paternal attachment and maternal attachment security are negatively linked to adolescents’ PIU. Moreover, the moderation analyses showed that higher levels of grit buffered against boys’ PIU in the context of paternal attachment security and girls’ PIU in the context of paternal attachment insecurity.

In accordance with prior research [17,18,19,20], attachment theory [14,15], and the first hypothesis, the current study highlights that parental attachment security plays a crucial role in mitigating the onset of PIU during adolescence. One possible explanation is that adolescents with parental attachment security are likely to have confidence in real interactions, developing trusting and stable peer relationships. In this context, they may not use the Internet as a virtual retreat to protect themselves from feelings of loneliness and fears about social interactions [53].

Furthermore, the current study shows that higher levels of grit buffered against boys’ PIU in the context of paternal attachment security and girls’ PIU in the context of paternal attachment insecurity. These findings are partially in line with the second hypothesis; namely, great grit enhances the negative association of paternal attachment with adolescents’ PIU (2b), but the opposite gender pattern is not supported [24]. These gender differentiation effects may be ascribed to traditional Chinese cultural values [21]: fathers play an essential role in disciplining their sons. Indeed, as highly emphasized in Chinese culture, the quality of father-child relations can set a masculine traits’ model for boys and cultivate their adaptive behavioral patterns [22]. Given this, fathers often spend relatively less time with their daughters, and thus positive, father-daughter relationships may have a compensatory influence on their adaptive behavioral patterns [21]. Apart from favorable father-son interactions, adolescent boys are also expected to be more psychologically firm and emotionally independent than girls in traditional Chinese culture [33]. In light of this cultural emphasis, high grit prevents adolescents from excessively using the Internet, and they focus on their academic assignments and pursue their long-term goals [31]. However, the buffering role of grit varies in different contexts of paternal attachment, depending on the adolescents’ gender.

Concerning covariates, past research on these topics has produced significant, but contradictory findings [34,35]. Distinct from previous studies, the current study does not reveal significant associations of age and family SES with adolescents’ PIU. One possible explanation could be related to the increasing trend of Internet use in Chinese adolescents. This is particularly true in the current sample as these adolescents were recruited from urban settings, in which Internet use is more universal for all ages and family backgrounds.

### 4.1. Theoretical and Practical Implications

According to these significant findings, the current study has several theoretical and practical implications. From a theoretical perspective, this study enriches the socio-ecological framework and attachment theory, as well as the pertinent literature, explaining the processes of the association between parental attachment and PIU in adolescence. In terms of practical implications, the following aspects should be considered. First, the current findings show the protective role of parental attachment security in adolescents’ PIU, highlighting the need for family-based interventions in adolescents’ Internet use. Give this, health care professionals and educators should guide parents to recognize the symptoms of their children’s PIU earlier and provide constructive recommendations to facilitate positive parent-child relationships in managing adolescents’ Internet use. Second, investigation into the risk and protective correlates of PIU could help health care professionals and educators identify groups of adolescents who are at a pronounced risk of PIU and require additional and professional assistance. For instance, in the context of paternal attachment insecurity, greater grit could help girls buffer against PIU. Therefore, facilitating grit training for girls would be one crucial intervention target. This is particularly salient in Chinese society, as paternal involvement in adolescent girls is relatively limited. By contrast, for boys, facilitating a positive father-son relationship and grit training are both indispensable.

### 4.2. Limitations and Recommendations for Future Research

Despite these meaningful implications, several limitations should be considered when interpreting the results. First, the current study is based on a cross-sectional design. Thus, we cannot exclude the inherent individual differences of the study participants, and we are unable to infer causality concerning the relationships observed between study variables. Given this, future studies should use a longitudinal design to examine how PIU changes over time and tease apart its associations with contextual and personal characteristics. Second, the current study relies solely on self-reported questionnaires, and thus, the findings may potentially be influenced by social desirability and common method bias. Prospective studies should consider using multiple methods and a multiple informant approach to balance these biases. Third, PIU is assessed using a questionnaire with a yes-no format in this study, which consequently reduced the variance of PIU. Although we adopted the original version of the questionnaire that has been used in prior research among Chinese adolescents, future studies may employ an ordinal response format to obtain a more comprehensive picture of PIU.

## 5. Conclusions

Using a large-scale sample of Chinese adolescents, the current study indicates that both paternal and maternal attachment security should be considered as one of the key protective factors in the onset of PIU and highlights the importance of differentiating adolescents’ gender and their grit levels in the attachment-PIU link. Specifically, the buffering role of grit in PIU varies by the levels of paternal attachment security, depending on adolescents’ gender.

## Figures and Tables

**Table 1:** Descriptive statistics and correlation analyses of the study variables for Chinese adolescents.

	Boys (*n* = 1164; 43.5%)			Girls (*n* = 1513; 56.5%)									
	*M*	*SD*	Range	*M*	*SD*	Range	*t*	1	2	3	4	5	6
1. Paternal Attachment	3.36	0.77	1–5	3.39	0.81	1–5	−1.09	−	0.70 ***	0.25 ***	−0.18 ***	−0.03	0.03
2. Maternal Attachment	3.49	0.73	1–5	3.60	0.80	1–5	−3.87 ***	0.71 ***	−	0.25 ***	−0.20 ***	0.01	0.04
3. Grit	3.23	0.64	1–5	3.14	0.66	1–5	3.51 ***	0.27 ***	0.27 ***	−	−0.32 ***	−0.06 *	0.07 **
4. Problematic Internet Use	2.94	2.56	0–8	2.49	2.35	0–8	4.72 ***	−0.24 ***	−0.22 ***	−0.29 ***	−	−0.03	−0.04
5. Age	15.66	1.58	13–18	15.68	1.56	13–18	−0.35	0.01	0.07 **	−0.09 ***	0.04	−	0.06 *
6. Socioeconomic Status	15.21	3.99	5–29	15.04	4.04	5–27	1.05	0.05 *	0.05 *	0.03	0.02	0.05 *	−

Notes: *N* = 2677. The standardized socioeconomic status score ranged from −8.92 to 13.66 for boys and from −8.92 to 11.52 for girls. Correlation coefficients displayed above the diagonal are for boys, below for girls. * *p* < 0.05 ** *p* < 0.01 *** *p* < 0.001.

**Table 2:** Regression analysis examining the associations of paternal attachment, gender, grit, and covariates with problematic Internet use (PIU).

Statistical Predictors	*b*	*SE b*	95% CI for *b*		*t*	*p*
Paternal Attachment	−0.42	0.06	−0.53	−0.30	−7.04	<0.001
Gender ^a^	−0.58	0.09	−0.76	−0.40	−6.30	<0.001
Grit	−1.03	0.07	−1.17	−0.89	−14.48	<0.001
Age	−0.02	0.03	−0.08	0.03	−0.74	0.46
Socioeconomic Status	0.01	0.01	−0.01	0.04	0.98	0.33
Paternal Attachment × Gender	−0.17	0.12	−0.40	0.06	−1.42	0.16
Paternal Attachment × Grit	−0.02	0.08	−0.17	0.13	−0.24	0.81
Gender × Grit	0.31	0.14	0.03	0.59	2.17	0.03
Paternal Attachment × Gender × Grit	0.38	0.15	0.09	0.68	2.53	0.01

Notes: *N* = 2677. *b* refers to the unstandardized beta. ^a^ Coded as 1 = boys, 2 = girls.

**Table 3:** Regression analysis examining the associations of maternal attachment, gender, grit, and covariates with PIU.

Statistical Predictors	*b*	*SE b*	95% CI for *b*		*t*	*p*
Maternal Attachment	−0.45	0.06	−0.57	−0.32	−7.21	<0.001
Gender ^a^	−0.53	0.09	−0.71	−0.35	−5.68	<0.001
Grit	−1.04	0.07	−1.18	−0.90	−14.48	<0.001
Age	−0.01	0.03	−0.07	0.05	−0.36	0.72
Socioeconomic Status	0.01	0.01	−0.01	0.04	0.98	0.33
Maternal Attachment × Gender	−0.01	0.12	−0.25	0.23	−0.09	0.93
Maternal Attachment × Grit	−0.03	0.08	−0.18	0.12	−0.40	0.69
Gender × Grit	0.27	0.14	−0.01	0.55	1.87	0.06
Maternal Attachment × Gender × Grit	0.29	0.16	−0.02	0.59	1.83	0.07

Notes: *N* = 2677. *b* refers to the unstandardized beta. ^a^ Coded as 1 = boys, 2 = girls.
